# Assessment of 19 Genes and Validation of CRM Gene Panel for Quantitative Transcriptional Analysis of Molecular Rejection and Inflammation in Archival Kidney Transplant Biopsies

**DOI:** 10.3389/fmed.2019.00213

**Published:** 2019-10-01

**Authors:** Tara Sigdel, Mark Nguyen, Juliane Liberto, Dejan Dobi, Henrik Junger, Flavio Vincenti, Zoltan Laszik, Minnie M. Sarwal

**Affiliations:** ^1^Department of Surgery, University of California, San Francisco, San Francisco, CA, United States; ^2^Department of Nephrology, University of California, San Francisco, San Francisco, CA, United States; ^3^Department of Pathology, University of California, San Francisco, San Francisco, CA, United States

**Keywords:** kidney transplantation, acute rejection, biomarker, transcriptomics analysis, FFPE

## Abstract

**Background:** There is an urgent need to develop and implement low cost, high-throughput standardized methods for routine molecular assessment of transplant biopsies. Given the vast archive of formalin-fixed and paraffin-embedded (FFPE) tissue blocks in transplant centers, a reliable protocol for utilizing this tissue bank for clinical validation of target molecules as predictors of graft outcome over time, would be of great value.

**Methods:** We designed and optimized assays to quantify 19 target genes, including previously reported set of tissue common rejection module (tCRM) genes. We interrogated their performance for their clinical utility for detection of graft rejection and inflammation by analyzing gene expression microarrays analysis of 163 renal allograft biopsies, and subsequently validated in 40 independent FFPE archived kidney transplant biopsies at a single center.

**Results:** A QPCR (Fluidigm) and a barcoded oligo-based (NanoString) gene expression platform were compared for evaluation of amplification of gene expression signal for 19 genes from degraded RNA extracted from FFPE biopsy sections by a set protocol. Increased expression of the selected 19 genes, that reflect a combination of specific cellular infiltrates (8/19 genes) and a graft inflammation score (11/19 genes which computes the tCRM score allowed for segregation of kidney transplant biopsies with stable allograft function and normal histology from those with histologically confirmed acute rejection (AR; *p* = 0.0022, QPCR; *p* = 0.0036, barcoded assay) and many cases of histological borderline inflammation (BL). Serial biopsy shaves used for gene expression were also processed for *in-situ* hybridization (ISH) for a subset of genes. ISH confirmed a high degree of correlation of signal amplification and tissue localization.

**Conclusions:** Target gene expression amplification across a custom set of genes can identify AR independent of histology, and quantify inflammation from archival kidney transplant biopsy tissue, providing a new tool for clinical correlation and outcome analysis of kidney allografts, without the need for prospective kidney biopsy biobanking efforts.

## Introduction

Renal transplantation is the preferred treatment of end stage renal disease (ESRD) (1, 2); however, acute rejection, both T cell mediated rejection (TCMR) or antibody-mediated rejection (AMR), remain barriers to long-term graft survival. Current classification methods of histological rejection by modified Banff classification, suffer from heterogeneities in biopsy sampling, poor correlations between blinded pathology reads, and an inability to accurately quantify the inflammatory burden in the allograft, which is an important predictor of long-term graft function and survival (3–12). Unbiased and quantitative measurement of inflammation in the graft and its evolution over time and with treatment is an important measure to guide immunosuppression delivery. Measuring molecular inflammation can also discern clinical variance in the histological injury of borderline (BL) characteristics in the kidney, a finding that is not always detrimental to the allograft, specifically if it is also molecularly quiescent (13–18).

The tissue common rejection module (tCRM) score is a computed algorithm to assess inflammatory score for acute rejection, consisting of 11 genes, originally identified and validated in over 700 transplant tissue microarrays from kidney, heart, lung, and liver transplants (5, 19). A tCRM gene expression score was identified on the microarray data and then validated by QPCR on RNALater collected kidney transplant needle biopsies that had been collected prospectively, as part of biobanking efforts (5). The tCRM gene expression score of kidney tissues collected at 6 months directly correlated with risk of progressive interstitial fibrosis and tubular atrophy on 24 months protocol biopsies (19). In order to allow this score to be assessed on archival FFPE tissue blocks, we developed and optimized a protocol that would allow for target gene analysis on degraded RNA (11).

In this report, we applied our protocol that measures gene expression of 19 genes from RNA isolated from four 10-micron shaves off an FFPE block with histologically confirmed different phenotypes of normal functioning (NL), T cell-mediated rejection (TCMR), antibody-mediated rejection (ABMR), borderline changes (BL), and polyoma virus nephropathy (PVAN). In addition, chromogenic *in situ* hybridization (cISH) was employed on assessing spatial expression of two of the most significant genes (CXCL9 and CXCL10) on serial sections to demonstrate biological relevance and accuracy of gene expression data on FFPE blocks.

## Methods

### Patient Enrollment and Study Design

One hundred sixty-three kidney transplant recipient biopsies, with and without clinical graft dysfunction and matched biopsy histology, were profiled by oligo-based microarrays and unsupervised sub-clustering performed across the 19 target genes. Forty independent renal transplant recipients were identified with Banff supported diagnosis of acute T cell-mediated rejection (TCMR, *n* = 8), antibody-mediated rejection (ABMR, *n* = 8), borderline changes (BL, *n* = 8), polyoma virus nephropathy (PVAN, *n* = 8), and normal functioning (NL, *n* = 8) were used for clinical validation (Table 1) (20–23). The basis of inclusion of TCMR, ABMR, BL, and PVAN was to evaluate heterogeneous injury types. Demographic information is provided in Table 1. The study was approved by the Institutional Review Board and Ethics Committee of the University of California, San Francisco (UCSF), CA. All patients provided written informed consent to participate in the research, in full adherence to the Declaration of Helsinki. The clinical and research activities being reported are consistent with the Principles of the Declaration of Istanbul as outlined in the Declaration of Istanbul on Organ Trafficking and Transplant Tourism.

**Table 1:** Demographic table.

**Pathology diagnosis**	**NL (*n* = 8)**	**BL (*n* = 8)**	**TCMR (*n* = 8)**	**ABMR (*n* = 8)**	**PVAN (*n* = 8)**	***P*-value**
Recipient age^*^ **(years)**	52.9 ± 13.9 (54.0; 37–74)	51.8 ± 11.6 (49.5; 40–74)	48.5 ± 14.9 (46.0; 27–75)	44.6 ± 14.0 (48.5; 17–58)	54.3 ± 13.9 (59.0; 32–69)	0.71 (ns)
**Gender (%M)**	50.0	50.0	50.0	75.0	75.0	0.65 (ns)
Time post-transplant^*^ **(months)**	6.1 ± 0.4 (6.0; 6–7)	6.6 ± 2.8 (6.0; 2–12)	7.6 ± 7.5 (6.0; 0.5–24)	91.6 ± 99.7 (48.0; 0.5–264)	34.3 ± 40.7 (17.5; 4–102)	0.004
eGFR^#^ (ml/min/1.73 m^**2**^)^*^	63.1 ± 12.4 (59.0; 48–87)	51.5 ± 18.4 (49.0; 27–79)	50.3 ± 20.7 (51.0; 21–87)	35.4 ± 23.3 (30.0; 9–69)	35.6 ± 13.1 (36.0; 21–55)	0.02
Creatinine (mg/dL)^*^	1.2 ± 0.2 (1.1; 0.9–1.5)	1.5 ± 0.5 (1.6; 0.8–2.1)	1.6 ± 0.9 (1.3; 0.6–3.3)	2.8 ± 2.1 (2.0; 1.02–6.62)	2.1 ± 0.6 (2.1; 1.36–3.06)	0.04
**Urine protein/Creatinine** (mg Pro/mg Cre)^*^	0.2 ± 0.0 (0.1; 0.11–0.2)	0.2 ± 0.0 (0.2; 0.11–0.26)	0.2 ± 0.2 (0.1; 0.08–0.53)	3.7 ± 4.6 (2.3; 0.14–14.3)	0.2 ± 0.1 (0.2; 0.08–0.32)	0.006
**Transplant type (%)**						0.22 (ns)
LRRT	25.0	12.5	12.5	50.0	12.5	
LURT	25.0	25.0	12.5	25.0	0.0	
DDRT	25.0	62.5	62.5	12.5	75.0	
SPK	25.0	0.0	12.5	12.5	0.0	
SHK	0.0	0.0	0.0	0.0	12.5	
**Recipient race (%)**						0.80 (ns)
Caucasian	50.0	50.0	25.0	50.0	50.0	
Hispanic/Latina	37.5	25.0	12.5	25.0	25.0	
Asian	0.0	25.0	25.0	12.5	25.0	
African American	12.5	0.0	25.0	0.0	0.0	
Hawaiian	0.0	0.0	12.5	0.0	0.0	
Other	0.0	0.0	0.0	12.5	0.0	
**Native renal disease (%)**						0.61 (ns)
Hypertension (HTN)	25.0	12.5	25.0	0.0	0.0	
Glomerulonephritis	0.0	12.5	25.0	37.5	12.5	
Type I diabetes (DBI)	25.0	0.0	12.5	12.5	0.0	
Type II diabetes (DBII)	12.5	25.0	12.5	0.0	25.0	
HTN + DBI/DBII	12.5	0.0	0.0	0.0	12.5	
Unknown	12.5	12.5	0.0	25.0	25.0	
Tubulointerstitial nephritis	12.5	0.0	0.0	0.0	12.5	
Polycystic kidney disease	0.0	37.5	12.5	25.0	12.5	
HIV	0.0	0.0	12.5	0.0	0.0	

*NL, normal graft function; BL, borderline changes; TCMR, T-cell mediated rejection; AMR, antibody mediated rejection; PVAN, polyomavirus; LRRT, living related renal transplant; LURT, living unrelated renal transplant; DDRT, deceased donor renal transplant; SPK, simultaneous pancreas-kidney transplant; SHK, simultaneous heart-kidney transplant*.

* *Unit listed: Mean ± SD (Median; Range), P-values for continuous values are calculated with 1 way ANOVA, and for categorical variables with Fisher Exact test*.

# *eGFR was calculated with Modification of Diet in Renal Disease (MDRD) Study equation (24)*.

### Histopathology

Tissue cores were fixed in 10% neutral buffered formalin and embedded in paraffin. First, 2 μm-thick consecutive sections were cut and stained with hematoxylin and eosin, periodic acid Schiff (PAS), and PAS-silver as part of the routine diagnostic workup. Immunofluorescence microscopy was performed on frozen sections with indirect method for complement 4d (C4d), and direct method for immunoglobulin G (IgG), IgM, IgA, C3, C1q, fibrinogen and albumin. Classification into TCMR, ABMR, BL, and NL were based on Banff classification (20–23). The diagnosis of PVAN required nuclear positivity in the tubular epithelium by SV-40 stain horseradish peroxidase-diaminobenzidine tetrahydrochloride detection method. Estimated glomerular rate (eGFR) was calculated using Modification of Diet in Renal Disease (MDRD) Study equation (24).

### Interrogation of Microarray Data for the 19 Genes Across Different Kidney Transplant Injury Phenotypes

Microarray data from 163 kidney transplant biopsies (GSE72925) was analyzed by unsupervised analysis for sample clustering across the 19 genes; the 11 CRM genes (BASP, CD6, CXCL10, CXCL9, INPP5D, ISG20, LCK, NKG7, PSMB9, RUNX3, TAP1) and additional eight immune cell specific genes relating to different cell types, such as overall leukocyte burden (CD45), B cells (CD20), T cells (CD4, CD8A, FOXP3), macrophages (CD68), endothelial cells (CD31, PECAM1), and collagen (COL4A1).

### Total RNA Extraction

We followed previously published protocol for the extraction of RNA from FFPE tissues (11). Based on our previous experience, we used 4 × 10 μm-thick sections to extract total RNA from FFPE samples. For rapid purification, PureLink FFPE Total RNA Isolation Kit was used (Invitrogen, Thermo Fischer Scientific, Foster City, CA) according to the manufacturer's recommendations. The RNA data quality was assessed by 260/280 absorption signal ratio and the RIN number.

### NanoString's Barcoded Oligos Design and Assay

Barcoded Codesets for each gene studied including 5 reference genes (GAPDH, GUSB, HPRT1, LDHA, and TBP) used in the study were designed by NanoString Technologies as a service (detail information available in Supplemental Table S1). The NanoString assay was performed (NanoString Technologies, Seattle, WA) as follows. Briefly, 50 ng of each RNA sample was added to the CodeSet in hybridization buffer and incubated at 65°C for 16 h. The CodeSet consisted of Reporter and Capture probes that hybridize to the target sequences of interest, forming a tripartite complex. After the assay, the raw counts for each assay were collected using the NanoString data analysis software, nSolver® (NanoString Technologies, Seattle, WA). Raw counts were derived from the RCC data files. Normalization of the data was performed using nSolver® for the following two methods. (1) Positive control normalization: gene expression data is normalized to the mean of the POS control probes for each assay. (2) RNA content normalization: gene expression data was normalized to the geometric mean of housekeeping genes in the CodeSet.

### cDNA Synthesis, and Gene Expression Quantification Using qPCR

A total of 50-ng RNA was reversed transcribed into complementary DNA (cDNA) using SuperScript VILO (Invitrogen, Thermo Fischer Scientific, Foster City, CA) and then amplified in a target specific amplification step for all 19 genes (above) using TaqMan PreAmp Master Mix and TaqMan Primers and Probes (Invitrogen, Thermo Fischer Scientific, Foster City, CA) (Supplemental Table S2) for a total of 18 amplification cycles. QPCR reactions were performed in the Fluidigm BioMark FD system using 18S gene as a housekeeping gene and Human XpressRef Universal Total RNA (Qiagen, Valencia, CA) as a reference RNA for 40 cycles. Resulting chip data was initially analyzed for quality control using the BioMark Analysis Software Version 2.0 (Fluidigm, South San Francisco, CA) and Ct values were exported into Excel. Normalization of the data was done in two steps. (1) Ct values of individual genes were normalized against Ct value of 18S for each gene to get dCt values. (2) dCt values of each sample was normalized against dCt values of the reference sample to get ddCt values which was subsequently used to calculate fold change (RQ) values for each gene in each sample.

### Statistical Data Analysis and Confounder Analysis

Because of its steady expression, 18S ribosomal RNA has been a popular reference RNA in gene expression analyses. For the QPCR platform, 18S ribosomal RNA was used as the reference gene. However, for the nCounter system of NanoString, the system could not handle high abundance of 18S ribosomal RNA. We chose 5 common reference RNAs (GUSB, HPRT1, LDHA, TBP, GAPDH) as reference RNAs and used mean signal as reference value. We tested gene expression correlation by comparing Ct values of 18S ribosomal RNA with mean Ct values of the 5 reference RNAs (GUSB, HPRT1, LDHA, TBP, GAPDH) using 10 FFPE samples. There was a strong correlation with Pearson correlation (r) of 0.97 (*R*^2^ = 0.87) (*P* < 0.0001) in between the Ct value of 18S ribosomal RNA and the mean Ct value of the five reference genes listed above. This demonstrated that gene expression analyses performed with either 18S ribosomal RNA or the 5 common reference genes are comparable. Following reference gene normalization, QPCR platform data was log2 transformed. Unsupervised and supervised hierarchical clustering was performed using GENE-E (https://software.broadinstitute.org/GENE-E/) with utilization of one minus Pearson correlation and average as the metric and linkage method, respectively. Correlation values were calculated using Pearson and Spearman rank-based correlation method (GraphPad Prism, La Jolla California USA, www.graphpad.com) where the correlation coefficient, *r*, ranges from −1 to +1. Significant difference in two sets of data was determined by unpaired *T*-test with two-tailed *P*-value option and with FDR correction for multiple testing using two-stage step-up method of Benjamini, Keieger, and Yekutieli (using GraphPad Prism). A value of <0.05 was considered significant. The relationship in between gene expression and the biopsy scores was calculated using The Jonkheere-Terpstra test. The Spearman rank-order correlation was performed to calculate rho.

As seen on Table 1, we balanced recipient age, gender, transplant type, recipient race, and native renal disease (ESRD). Other parameters, such as serum creatinine, eGFR, and proteinuria were significantly different among injury phenotypes, which was expected. Post-transplant times were significantly different among different injury phenotypes (*p* = 0.004). A confounder analysis on gene expression levels of tCRM genes and months post-transplantation resulted in a *p*-value of 0.32 demonstrating post-transplantation time was not a factor driving gene expression values.

### Chromogenic ISH (CISH) Assay for CXCL9 and CXCL10

The RNAscope platform (ACD Bio, Newark, CA) was used to quantify gene transcripts *in situ*. Probes against two of the most significant genes in rejection, and with readily available commercial assays, namely CXCL9 (cat # 440161) and CXCL10 (cat # 311851) (ACD Bio, Newark, CA) were applied on consecutive 2 μm-thick FFPE tissue sections and were detected by alkaline-phosphatase-based technique (RNAscope 2.5 HD Assay—Red) coupled with Warp Red chromogen (Biocare Medical, Pacheco, CA) followed by hematoxylin counterstain. Depending on the abundance and distribution of the transcript, the positive signal can be seen as separate dots or fused group(s) of multiple dots. After detection, the tissue sections were digitized by Aperio ScanScope XT. Whole-slide digital images were analyzed by the Definiens Tissue Studio platform's Dot Count module. In brief, the software models an “average” cell based on the hematoxylin counterstain first, and assigns each dot to a particular cell and counts them. Thresholds were adjusted individually and accuracy of the settings was checked by evaluation of 12 randomly selected HPFs at 40X. The number of dots in the entire section/1,000 cell characterized the expression level of a given gene.

## Results

### Acute Rejection, IFTA, and PVAN Share Signals of Molecular Inflammation in Microarray Datasets From 163 Kidney Transplant Biopsies

**Figure 1:**
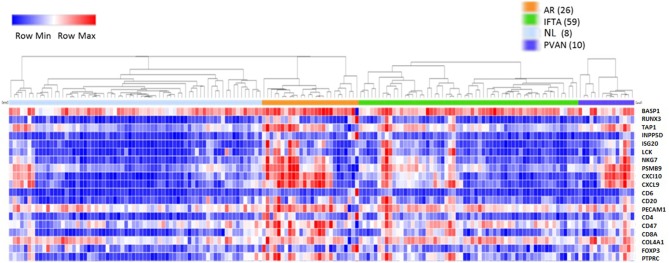
Heterogeneity of kidney graft injury across different injury phenotypes. As anticipated, CRM genes and genes specific to immune related genes are upregulated in the tissue from recipients of kidney transplantation with AR episodes. In addition, increased gene expression is seen with PVAN and IFTA subtypes. Furthermore, some tissues from normal graft biopsies also had molecular signatures that correspond to inflammation.

Initially, to evaluate the performance of the gene expression of the selected 19 genes across different transplant injury phenotypes we evaluated unsupervised interrogation of sample clustering across the 19 genes from the microarray data generated on 163 kidney tissue samples (68 NL, 26 AR, 10 PVAN, and 59 IFTA) (GEO Accession: GSE72925). As seen in Figure 1, there was an overall increased expression of most of these genes in AR, supporting the selection of these markers for rejection detection. The gene expression in other injury phenotypes including PVAN, IFTA, and histological and clinically stable biopsies (NL) is seen in Figure 1, highlighting that molecular inflammation is seen in biopsies not classified as AR by histology. This observation is in keeping with other studies that have reported that molecular inflammation in the graft can be sub-clinical and not always congruent with histology (25).

### Validation of Perturbation of Transcriptional Signals by Either Fluidigm or NanoString Methods in FFPE Sections in Different Transplant Injury Phenotypes

**Figure 2:**
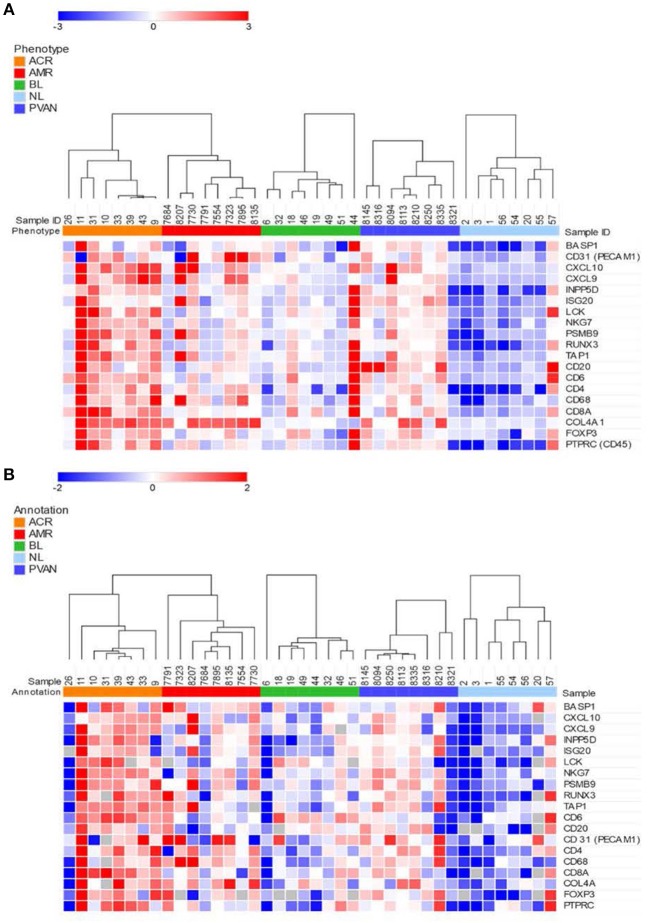
Quantitative methods of gene expression of CRM genes and immune-related genes differentiate kidney transplant biopsies with different transplant injuries. Expression of the genes across 40 unique samples that included 8 NL, 8 TCMR, 8 ABMR, 8 BL, and 8 PVAN. **(A)** As quantified by NanoString's gene expression platform. **(B)** As quantified by QPCR (Fluidigm).

We evaluated gene expression changes in different kidney transplant phenotypes on 40 independent samples (8 NL, 8 TCMR, 8 ABMR, 8 BL and 8 PVAN) using two platforms commonly used for quantification of gene expression. Gene expression data from the NanoString (barcoded oligos) platform showed significant increases in gene expression level in rejection (TCMR and ABMR) phenotypes compared to other phenotypes (including BL, PVAN, and NL) (Figure 2A). Thirteen of the 19 genes tested (BASP1, CXCl10, CXCL9, ISG20, LCK, NKG7, PSMB9, TAP1, CD31, CD4, CD68, COL4A, PTPRC) showed significantly increased mRNA levels in injury phenotypes (TCMR, ABMR, BL, and PVAN) when compared to NL (*p* ≤ 0.05). More genes were significantly increased in TCMR (BASP1, CXCL10, CXCL9, INPP5D, ISG20, LCK, RUNX3, CD6, CD4, COL4A) than in ABMR (INPP5D, ISG20, NKG7, RUNX3, CD31, CD4, CD68, COL4A) when compared to NL with *p*-value ≤0.05, highlighting subtle differences in tissue inflammatory components between TCMR and ABMR.

Similar to the NanoString data, gene expression data from the QPCR platform showed an overall increased gene expression level in AR (TCMR and ABMR) phenotypes compared to other phenotypes (including BL, PVAN, and NL) (Figure 2B). 7 of the 19 genes assayed (CXCL10, CXCL9, ISG20, LCK, RUNX3, CD20, CD247) showed an increased mRNA level in injury phenotypes (TCMR, ABMR, BL, and PVAN) when compared to NL (*p* ≤ 0.05). On individual gene level, mRNA transcripts of CXCL10, LCK, and TAP1 were significantly increased in TCMR compared to NL (*p* ≤ 0.05). mRNA transcripts of CD68 and COL4A were increased in ABMR compared to NL (*p* ≤ 0.05) (Supplemental Table S3).

### The tCRM Gene Composite Score Is Specifically Increased in Acute Rejection, for Both TCMR and ABMR

**Figure 3:**
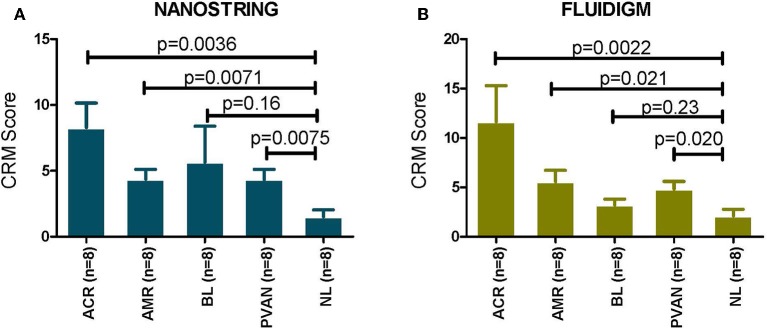
CRM scores are significantly increased in transplant injury. CRM score was used to evaluate difference among different transplant injury phenotypes. **(A)** The CRM scores calculated from the NanoString (barcoded oligos) data for injury phenotypes (TCMR, AMR, PVAN) were significantly higher than the CRM scores for NL phenotypes (*p* ≤ 0.05). **(B)** The CRM scores calculated from the QPCR data for injury phenotypes (TCMR, AMR, PVAN) were significantly higher than the CRM scores for NL phenotypes (*p* ≤ 0.05). Even though there was a trend of higher CRM scores for borderline changes (BL), they were not significant (*p* > 0.05) for both platforms.

In the datasets generated by both the platforms used, the CRM scores for injury phenotypes (TCMR, ABMR, and PVAN) were significantly higher than the CRM scores for NL phenotypes (*p* ≤ 0.05). Even though there was a trend of higher CRM scores for borderline changes (BL), overall these were significantly lower than those in AR (Figure 3).

We also evaluated association (using Jonkheere-Terpstra test) with and correlation (using Spearman's rank-order) of immune injury related genes' expression by both the platforms with biopsy i-score (interstitial inflammation). With NanoString gene expression data, the expression of CD45 (*p* = 0.006, rho = 0.41), CD68 (*p* = 0.0001, rho = 0.57), CD4 (*p* = 1.1E-05, rho = 0.62), and CD8A (*p* = 1.34E-06, rho = 0.68) were all correlated with i-score significantly. With QPCR gene expression data, the expression of CD45 (*p* = 8.53E-05, rho = 0.54), CD68 (*p* = 3.27E-05, rho = 0.60), CD4 (*p* = 8.53E-05, rho = 0.54), and CD8A (*p* = 0.0003, rho = 0.52) were all correlated with i-score significantly. Gene expression of COL4A gene was significantly associated with ct (tubular atrophy) and ci (interstitial fibrosis) scores with NanoString data (ct, *p* = 0.05, rho = 0.27, ci, *p* = 0.03, rho = 0.30) and QPCR (ct, *p* = 0.006, rho = 0.39, ci, *p* = 0.002, rho = 0.46). This demonstrated the potential usefulness of the molecular scores in clinical management of the kidney graft.

### Strong Correlation in Between Chromogenic ISH Signal of CXCL9 and CXCL10 Gene-Transcripts With Gene Expression Data

**Figure 4:**
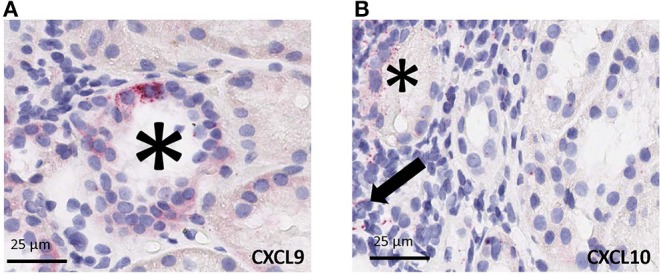
Cell specific gene expression of CXCL9 and CXCL10 by chromogenic *in situ* hybridization (ciSH) data agrees with gene expression data on bulk tissue. Representative images from biopsy samples with T-cell mediated rejection. Chromogenic *in situ* hybridization for CXCL9 **(A)** and CXCL10 **(B)** shows high-level expression on tubular epithelial cells (asterisks) and some scattered mononuclear cells (arrow), 400×. Rare signal was noted in some glomeruli, however no definite signal was detected in the vascular compartment or in the interstitium other than the inflammatory cells (32, 33).

Using RNAscope platform by ACD bio, we quantified gene transcripts of two genes that are highly expressed in infiltrating lymphocytes, for CXCL9 and CXCL10, *in situ*. Depending on the abundance and distribution of the transcript, the positive signal can be seen as separate dots or fused group(s) of multiple dots (as seen in the representative images from biopsy samples with T-cell mediated rejection). Chromogenic *in situ* hybridization for CXCL9 and CXCL10 identified tubular epithelial cells as the primary source of these chemokines (Figure 4A) while scattered mononuclear cells also showed some expression (Figure 4B). In addition, rare signal was noted in some glomeruli; however, no definite signal was detected in the vascular compartment or in the interstitium other than the inflammatory cells.

**Figure 5:**
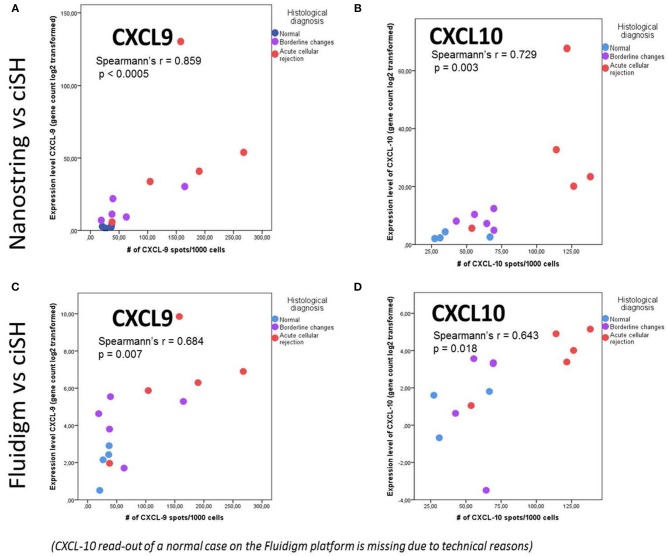
Chromogenic *in situ* hybridization (ciSH) data correlates with gene expression data on bulk tissue assessed by both Fluidigm and NanoString. We observed a strong correlation between CXCL9 and CXCL10 cISH spot count and the corresponding gene expression data when applied to a subset of 14 cases [NL (*n* = 4), BL (*n* = 5), and ACR (*n* = 5)] assessed with NanoString **(A,B)** and Fluidigm **(C,D)**. The X- and Y-axis values are different in case of NanoString and Fluidigm because of the different scale of the gene expression values.

The same FFPE block of a given case was used for both total RNA isolation and CISH. CISH data for CXCL9 with spot count/1,000 cells showed a strong correlation with the corresponding gene expression levels on both the NanoString platform (*r* = 0.859, *p* < 0.0005) (Figure 5A) and the Fluidigm QPCR platform (*r* = 0.684, *p* = 0.007) (Figure 5C). CISH data for CXCL10 with spot count/1,000 cells also showed a strong correlation with the corresponding gene expression levels on both the NanoString platform (*r* = 0.729, *p* < 0.003) (Figure 5B) and the QPCR Fluidigm platforms (*r* = 0.643, *p* = 0.018) (Figure 5D).

## Discussion

Molecular quantification of the overall inflammatory burden in the renal allograft is essential to establish at the time of an invasive biopsy. Our proposed method facilitates data collection to real-time results (overall experiment times are 4–6 h for the assays shown) and removes the burden of personnel needed for onsite prospective biobanking needing immediate tissue preservation in RNAlater. As a major benefit, we can now process a valuable clinical set of 19 genes for quantification of tissue inflammation and AR, using minimal tissue, from archived FFPE blocks, preserving most of the parent FFPE block for additional histologic analyses. Understanding the burden of inflammation in an allograft is critical for optimization of therapy, following response to chosen interventions and as a means to predict risk stratification for progressive chronic injury and allograft loss, when persistent. This study confirms that molecular profiling provides an objective assessment of graft inflammation, which can be a very valuable endpoint for observational and interventional clinical trials (26–28). The recognized difficulty in capturing and quantifying subtle differences by the histological scoring system can thus be aided by including a quantitative molecular scoring system on the same archival biopsy section.

QPCR provides an accurate assessment of gene transcripts in biological samples, such as blood, biopsy or urine (4, 6, 11, 29–31). In this study we show that multiplex high-throughput PCR by Fluidigm or a synthetic oligo based NanoString platform can further minimize sample input and handle low quality degraded RNA to amplify a robust set of 19 genes that can be computed to quantitate the inflammatory burden in the renal allograft (11). The parallel experiments on serially cut sections of kidney biopsies demonstrate that there is a strong correlation of gene expression *in situ* of prominent infiltrating lymphocyte markers (CXCL9 and CXCL10) in terms of CISH score with gene expression data from homogenized tissues. CXCL9 and CXCL10 were highly expressed in renal tubules during ACR, and were also found in infiltrating leukocytes in accordance with the spatial distribution pattern reported previously in a non-human primate model (32), and human renal transplant biopsies (33, 34). Strong correlation of gene expression data on serially sectioned bulk tissue with CISH data provides support of the biological relevance of the transcriptomic studies on bulk tissues based on FFPE specimens. This suggests that transcriptomic profiles preserved in FFPE blocks are both biologically relevant and clinically beneficial.

We acknowledge that the impact of this study would be further strengthened by the application of this technology to a larger number of samples and from its application in other study groups; the adoption of this assay as a primary endpoint for ongoing randomized clinical trial (RCT) is expected to provide additional clinical utility of this assay over time. Nevertheless, the development of the FFPE section processing protocol, its reduction to multiplexed PCR, and the validation of a composite tCRM score as a clinical surrogate endpoint for rejection and projection of future chronic injury decline, make strong arguments for this approach to benefit transplant patients and studies.

## Data Availability Statement

All datasets generated for this study are included in the manuscript/Supplementary Files.

## Ethics Statement

The studies involving human participants were reviewed and approved by Institutional Review Board and Ethics Committee of the University of California, San Francisco, CA. The patients/participants provided their written informed consent to participate in this study.

## Disclosure

Founder, KITBio, Organ-I; Past/Present Consulting: Bristol Meyers Squibb, Natera, Genentech, Novartis, Astellas; Treasurer, The Transplantation Society.

## Author Contributions

TS participated in designing the assays, supervising the data generation, analyzing the data, and writing the manuscript. MN participated in data analysis for gene expression assays and contributed in writing the manuscript. DD participated in tissue processing, CISH assays, data analysis for CISH, and contributed in manuscript writing. HJ participated in tissue processing, CISH assays, and data analysis for CISH. JL contributed in gene expression data generation, organization, and manuscript writing. FV provided the study samples, participated in discussions in data analysis strategies, and manuscript writing. ZL was part of the study design, contributed in tissue processing, supervision of CISH assay optimization, data analysis, and manuscript writing. MS contributed in study design, provided supervision on data generation and analysis, data reporting, and manuscript writing.

### Conflict of Interest

The authors declare that the research was conducted in the absence of any commercial or financial relationships that could be construed as a potential conflict of interest.
