# Supplementary material for: Assessment of 19 Genes and Validation of CRM Gene Panel for Quantitative Transcriptional Analysis of Molecular Rejection and Inflammation in Archival Kidney Transplant Biopsies
Source: Front Med (Lausanne). 2019 Oct 1;6:213. doi: 10.3389/fmed.2019.00213 (PMC6781675; doi:10.3389/fmed.2019.00213)
Supplement: Supplementary file 1 [file Table_1.DOCX]

**Supplemental Table S1:** NanoString codeset information

| S. No. | Vendor | NanoString ID reference | Accession | Position | Gene Symbol |
| --- | --- | --- | --- | --- | --- |
| 1 | NanoString | NM_006317.3:1160 | NM_006317.3 | 1161-1260 | BASP1 |
| 2 | NanoString | NM_000616.4:975 | NM_000616.4 | 976-1075 | CD4 |
| 4 | NanoString | NM_001254751.1:1722 | NM_001254751.1 | 1723-1822 | CD6 |
| 5 | NanoString | NM_001251.2:1140 | NM_001251.2 | 1141-1240 | CD68 |
| 6 | NanoString | NM_001768.5:1320 | NM_001768.5 | 1321-1420 | CD8A |
| 7 | NanoString | NM_001845.4:780 | NM_001845.4 | 781-880 | COL4A1 |
| 8 | NanoString | NM_001565.2:461 | NM_001565.2 | 462-561 | CXCL10 |
| 9 | NanoString | NM_002416.1:1975 | NM_002416.1 | 1976-2075 | CXCL9 |
| 10 | NanoString | NM_014009.3:1230 | NM_014009.3 | 1231-1330 | FOXP3 |
| 11 | NanoString | NM_005541.3:4075 | NM_005541.3 | 4076-4175 | INPP5D |
| 12 | NanoString | NM_002201.5:864 | NM_002201.5 | 865-964 | ISG20 |
| 13 | NanoString | NM_005356.2:1260 | NM_005356.2 | 1261-1360 | LCK |
| 14 | NanoString | NM_152866.2:620 | NM_152866.2 | 621-720 | CD20 |
| 15 | NanoString | NM_005601.3:632 | NM_005601.3 | 633-732 | NKG7 |
| 16 | NanoString | NM_000442.3:1365 | NM_000442.3 | 1366-1465 | CD31 (PECAM1) |
| 17 | NanoString | NM_002800.4:455 | NM_002800.4 | 456-555 | PSMB9 |
| 18 | NanoString | NM_080923.2:154 | NM_080923.2 | 155-254 | PTPRC (CD45) |
| 19 | NanoString | NM_004350.1:2085 | NM_004350.1 | 2086-2185 | RUNX3 |
| 20 | NanoString | NM_000593.5:2075 | NM_000593.5 | 2076-2175 | TAP1 |
| 11 | NanoString | NM_002046.5:1041 | NM_002046.5 | 1042-1141 | GAPDH* |
| 12 | NanoString | NM_000181.1:1350 | NM_000181.1 | 1351-1450 | GUSB* |
| 13 | NanoString | NM_000194.1:240 | NM_000194.1 | 241-340 | HPRT1* |
| 17 | NanoString | NM_001165414.1:1690 | NM_001165414.1 | 1691-1790 | LDHA* |
| 25 | NanoString | NM_001172085.1:587 | NM_001172085.1 | 588-687 | TBP* |

*Reference gene
