# Supplementary material for: Assessment of 19 Genes and Validation of CRM Gene Panel for Quantitative Transcriptional Analysis of Molecular Rejection and Inflammation in Archival Kidney Transplant Biopsies
Source: Front Med (Lausanne). 2019 Oct 1;6:213. doi: 10.3389/fmed.2019.00213 (PMC6781675; doi:10.3389/fmed.2019.00213)
Supplement: Supplementary file 2 [file Table_2.DOCX]

**Supplemental Table S2:** Primer information for primers used for QPCR assay

| S. No. | Vendor | Catalog no. | Item no. | Gene Symbol |
| --- | --- | --- | --- | --- |
| 1 | Thermo Fisher Scientific | 4331182 | Hs00234720_g1 | BASP1 |
| 2 | Thermo Fisher Scientific | 4331182 | Hs00300643_m1 | CD20 |
| 3 | Thermo Fisher Scientific | 4331182 | Hs00169777_m1 | CD31 (PECAM1) |
| 4 | Thermo Fisher Scientific | 4331182 | Hs01065472_m1 | CD4 |
| 5 | Thermo Fisher Scientific | 4331182 | Hs00198752_m1 | CD6 |
| 6 | Thermo Fisher Scientific | 4331182 | Hs00154355_m1 | CD68 |
| 7 | Thermo Fisher Scientific | 4331182 | Hs00233520_m1 | CD8A |
| 8 | Thermo Fisher Scientific | 4351372 | Hs01007468_m1 | COL4A1 |
| 9 | Thermo Fisher Scientific | 4331182 | Hs00171042_m1 | CXCL10 |
| 10 | Thermo Fisher Scientific | 4331182 | Hs00171065_m1 | CXCL9 |
| 11 | Thermo Fisher Scientific | 4331182 | Hs01085834_m1 | FoxP3 |
| 12 | Thermo Fisher Scientific | 4331182 | Hs00183290_m1 | INPP5D |
| 13 | Thermo Fisher Scientific | 4331182 | Hs00158122_m1 | ISG20 |
| 14 | Thermo Fisher Scientific | 4331182 | Hs00178427_m1 | LCK |
| 15 | Thermo Fisher Scientific | 4331182 | Hs01120688_g1 | NKG7 |
| 16 | Thermo Fisher Scientific | 4331182 | Hs04189704_m1 | PRPRC (CD45) |
| 17 | Thermo Fisher Scientific | 4331182 | Hs00544762_m1 | PSMB9 |
| 18 | Thermo Fisher Scientific | 4331182 | Hs00231709_m1 | RUNX3 |
| 19 | Thermo Fisher Scientific | 4331182 | Hs00388675_m1 | TAP1 |
| 20 | Thermo Fisher Scientific | 4331182 | Hs03003631_g1 | 18S* |

*Reference gene
