# Supplementary material for: Assessment of 19 Genes and Validation of CRM Gene Panel for Quantitative Transcriptional Analysis of Molecular Rejection and Inflammation in Archival Kidney Transplant Biopsies
Source: Front Med (Lausanne). 2019 Oct 1;6:213. doi: 10.3389/fmed.2019.00213 (PMC6781675; doi:10.3389/fmed.2019.00213)
Supplement: Supplementary file 3 [file Table_3.DOCX]

**Supplemental Table S3. Gene expression data on CRM gene set and immune-related genes with NanoString and QPCR platforms**

| **NanoString** | **Gene-Set** | **P-values** | **TCMR vs NR** | **ABMR vs NR** | **AR vs NR** | **PVAN vs NR** | **BL VS NR** | **IFTA vs NR** |
| --- | --- | --- | --- | --- | --- | --- | --- | --- |
|  | **CRM-set of genes** | **BASP1** | **0.024** | 0.0550 | **0.053** | **0.012** | 0.276 | **0.038** |
|  |  | **CXCL10** | **0.002** | 0.220 | **0.046** | **0.036** | **0.050** | **0.016** |
|  |  | **CXCL9** | **0.007** | 0.135 | **0.048** | 0.066 | **0.002** | **0.001** |
|  |  | **INPP5D** | **0.0002** | **0.016** | 0.165 | **0.007** | 0.322 | 0.123 |
|  |  | **ISG20** | 0.066 | **0.041** | 0.080 | **0.002** | 0.205 | **0.0004** |
|  |  | **LCK** | 0.006 | 0.406 | **0.021** | 0.154 | 0.229 | **0.042** |
|  |  | **NKG7** | 0.072 | **0.009** | 0.134 | **0.024** | 0.156 | **0.005** |
|  |  | **PSMB9** | 0.089 | 0.090 | **0.049** | **0.016** | **0.009** | **0.005** |
|  |  | **RUNX3** | **0.004** | **0.053** | 0.093 | **0.022** | 0.301 | 0.073 |
|  |  | **TAP1** | 0.077 | 0.098 | **0.016** | **0.020** | 0.086 | **0.001** |
|  |  | **CD6** | **0.011** | 0.742 | 0.491 | 0.237 | 0.322 | 0.111 |
|  | **Additional immune -elated genes** | **CD20** | 0.152 | 0.752 | 0.460 | **0.051** | 0.354 | 0.176 |
|  |  | **CD31 (PECAM1)** | 0.090 | **0.034** | 0.098 | **0.018** | 0.132 | **0.001** |
|  |  | **CD4** | **0.029** | **0.026** | 0.101 | 0.060 | 0.214 | **0.006** |
|  |  | **CD68** | 0.109 | **0.015** | **0.004** | **0.001** | 0.276 | **0.005** |
|  |  | **CD8A** | 0.207 | 0.062 | 0.186 | 0.087 | 0.293 | 0.062 |
|  |  | **COL4A** | 0.052 | **0.001** | 0.116 | **0.021** | 0.479 | **0.0001** |
|  |  | **FOXP3** | 0.085 | 0.298 | 0.163 | 0.174 | 0.345 | 0.232 |
|  |  | **PTPRC** | **0.005** | 0.105 | 0.243 | **0.050** | 0.227 | **0.016** |
| **QPCR** | **Gene-Set** | **P-values** | **TCMR vs NR** | **ABMR vs NR** | **AR vs NR** | **PVAN vs NR** | **BL VS NR** | **IFTA vs NR** |
|  | **CRM-set of genes** | **BASP1** | 0.162 | 0.162 | **0.053** | 0.477 | 0.907 | 0.112 |
|  |  | **CXCL10** | **0.004** | 0.196 | **0.046** | **0.017** | 0.056 | **0.016** |
|  |  | **CXCL9** | 0.149 | 0.225 | **0.048** | **0.051** | **0.023** | **0.022** |
|  |  | **INPP5D** | 0.220 | 0.293 | 0.165 | 0.301 | 0.618 | 0.171 |
|  |  | **ISG20** | 0.202 | 0.133 | 0.080 | 0.059 | 0.471 | **0.028** |
|  |  | **LCK** | **0.014** | 0.355 | **0.021** | 0.227 | 0.076 | **0.022** |
|  |  | **NKG7** | 0.204 | 0.061 | 0.134 | **0.017** | 0.179 | 0.074 |
|  |  | **PSMB9** | 0.162 | 0.171 | **0.049** | **0.003** | **0.037** | **0.017** |
|  |  | **RUNX3** | 0.067 | 0.293 | 0.093 | 0.490 | 0.983 | 0.258 |
|  |  | **TAP1** | **0.003** | 0.190 | **0.016** | 0.063 | 0.542 | **0.005** |
|  |  | **CD6** | 0.113 | 0.604 | 0.491 | 0.445 | 0.662 | 0.762 |
|  | **Additional immune-related genes** | **CD20** | 0.267 | 0.953 | 0.460 | 0.546 | 0.995 | 0.549 |
|  |  | **CD31 (PECAM1)** | 0.240 | 0.249 | 0.098 | 0.132 | 0.589 | 0.066 |
|  |  | **CD4** | 0.177 | 0.076 | 0.101 | **0.047** | 0.971 | 0.057 |
|  |  | **CD68** | 0.116 | **0.016** | **0.004** | **0.020** | 0.294 | **0.001** |
|  |  | **CD8A** | 0.213 | 0.403 | 0.186 | 0.366 | 0.499 | 0.156 |
|  |  | **COL4A** | 0.240 | **0.008** | 0.116 | 0.060 | 0.213 | 0.068 |
|  |  | **FOXP3** | 0.189 | 0.468 | 0.163 | 0.511 | 0.721 | 0.268 |
|  |  | **PTPRC** | 0.123 | 0.995 | 0.243 | 0.845 | 0.714 | 0.499 |

*Summary: Overall, both platforms showed comparable results with NanoString slightly better in distinguishing injuries on individual gene level. Thirteen out of the 19 genes by NanoString and 7 of the 19 genes by QPCR showed significantly increased mRNA levels in injury phenotypes when compared to NL (p≤0.05).*
